# ACE-Inhibitory Activity of Whey Proteins Fractions Derived of Fermentation by *Lacticaseibacillus rhamnosus* GG and *Streptococcus thermophilus* SY-102

**DOI:** 10.3390/foods12122416

**Published:** 2023-06-20

**Authors:** Laura Berenice Olvera-Rosales, Emmanuel Pérez-Escalante, Araceli Castañeda-Ovando, Elizabeth Contreras-López, Alma Elizabeth Cruz-Guerrero, Patricia Regal-López, Alejandra Cardelle-Cobas, Luis Guillermo González-Olivares

**Affiliations:** 1 Área Académica de Química, Ciudad del Conocimiento, Universidad Autónoma del Estado de Hidalgo, Mineral de la Reforma, Hidalgo 420390, Mexico; ol232998@uaeh.edu.mx (L.B.O.-R.); emmanuel_perez@uaeh.edu.mx (E.P.-E.); ovandoa@uaeh.edu.mx (A.C.-O.); elizac@uaeh.edu.mx (E.C.-L.); 2 Departamento de Biotecnología, División de Ciencias Biológicas y de la Salud, Unidad Iztapalapa, Universidad Autónoma Metropolitana, Ciudad de México 09340, Mexico; aec@xanum.uam.mx; 3 Laboratorio de Higiene, Inspección y Control de Alimentos, Departamento de Química Analítica, Nutrición y Bromatología, Campus Terra, Universidade da Santiago de Compostela, 27002 Lugo, Spain; patricia.regal@usc.es

**Keywords:** probiotic, whey fermentation, ACE inhibition, antihypertensive peptides, bioactive peptides

## Abstract

Many studies have reported the benefits of probiotic microorganisms and the production of angiotensin-converting enzyme (ACE) inhibitors. Determining the proteolytic and ACE inhibition capacities during whey fermentation was the goal of the study. *Lacticaseibacillus rhamnosus* GG, *Streptococcus thermophilus* SY-102, and both bacteria together were initially inoculated into whey, reaching an initial concentration of 10^8^ CFU per milliliter in each fermentation system. Through the use of TNBS, SDS-PAGE, and SEC-HPLC methods, the proteolytic profile was examined. An in vitro investigation was performed to test the ACE inhibition capacity. With *S. thermophilus*, the logarithmic phase of microbial development was shorter than with *L. rhamnosus* (6 and 12 h, respectively). The logarithmic phase in the co-culture fermentation, however, was extended to 24 h. There were no significant differences in pH between the fermentations. However, the co-culture had a greater concentration of protein hydrolysis (453 ± 0.06 μg/mL), as indicated by the amount of free amino groups. Similarly, this fermentation produced more low molecular weight peptides. The higher inhibition activity, which increased at the conclusion of the fermentation with the co-culture and reached 53.42%, was influenced by the higher peptide synthesis. These findings highlighted the significance of creating useful co-culture products.

## 1. Introduction

After casein coagulation, whey, a byproduct of the dairy industry, is removed during the cheesemaking procedure [1]. It is estimated that more than 160 million tonnes of whey are wasted annually in the word. Only a small portion, however, is processed to produce other by-products [2]. Because it poses a severe environmental problem, the elimination of this waste has become an ongoing challenge for the industry [2,3].

According to numerous studies, whey proteins are frequently a potential source of bioactive peptides, which have beneficial effects on human health [4,5]. However, these sequences need to be released following protein hydrolysis to have a biological impact [6]. Due to its effectiveness and profitability, microbial fermentation is one of the traditional processes used to produce peptides [7,8,9,10]. In this way, peptide sequences with various biological activities have been obtained by using a variety of microbial strains [11]. As a result, these microorganisms’ proteolytic mechanism encourage the release of peptide sequences [12].

Often peptides exert their effects through a variety of different mechanisms, one of which is their antihypertensive activity linked to the inhibition of angiotensin-converting enzyme (ACE) [13,14]. Angiotensin I is converted into angiotensin II by this enzyme which is a potent vasoconstrictor and the key regulator of blood pressure [15]. The description of sequences that inhibit ACE activity and hence produce an antihypertensive impact has been made possible by research into bioactive peptides [16]. Since whey was fermented with *L. rhamnosus* GG and *S. thermophilus* SY-102 in monoculture and with both bacteria for co-culture fermentation, the study’s goal was to examine the synthesis of low molecular weight peptides and the inhibitory potential of ACE.

## 2. Materials and Methods

### 2.1. Culture Preparation

Bacteria were collected from Iztapalapa campus of the Universidad Autónoma Metropolitana’s Food Biotechnology Laboratory. They were cultured in MRS broth and incubated at 42 °C for 24 h. Then, 9 mL of powdered whey solution (10% (*w*/*v*) obtained from Dairy Gold Co-Operative Society Ltd., Cork, Ireland) that had previously been heat-treated at 90 °C during 10 min were added to one milliliter of the inoculated broth. At 42 °C, it was incubated for 24 h. An amount of 100 mL of a 10% (*w/v*) powdered whey solution that had been pasteurized (90 °C for 10 min) received 1 mL of this solution. In the same way, each microorganism underwent independent conditioning. The solution was cooled down after 24 h of incubation at 42 °C. The solution obtained was the starter. A viable count was carried out to ascertain the initial bacterial concentration before the fermentations.

### 2.2. Fermentation

Three systems—inoculation with *Lacticaseibacillus rhamnosus* GG, inoculation with *Streptococcus thermophilus* SY-102, and finally inoculation with both microorganisms—were set up for fermentation. Each microorganism was inoculated into each system to reach initially about 1 × 10^8^ CFU per milliliter of bacteria calculated from the starter culture. In the co-culture case, the initial count of bacteria was inoculated equally (to reach 1 × 10^4^ CFU per milliliter of each microorganism). Whey powder containing 10% (*w/v*) was used to make the fermentation solution. Prior to inoculation, this solution was heat-treated at 90 °C during 10 min. A total of 48 h were spent incubating the samples at 42 °C. Aliquots were obtained every three hours and seeded on MRS agar plates using the microdrop technique to test the vitality of the microorganisms [17]. The samples were diluted from 1 × 10^−1^ to 1 × 10^−8,^ incubating for 48 h at 42 °C. Using an Eppendorf centrifuge, samples were spun at 24,600× *g* at 4 °C for 10 min to separate both coagulated proteins and biomass. The centrifuged samples’ supernatants were kept in a freezer at −4 °C for further examination. Each sample was examined three times.

### 2.3. Proteolytic Profile Analysis

By means of three distinct experiments, the proteolytic profile was characterized. Peptides concentration was determined using the technique of TNBS, and the peptides released during fermentation were separated using Tris-Tricine polyacrylamide gel electrophoresis (SDS-Tris-Tricine-PAGE). Finally, an HPLC analysis utilizing a size exclusion column was performed to detect the presence of peptides, particularly low molecular weight peptides.

#### 2.3.1. Analysis of Free Amino Groups

Using the 2,4,6-trinitrobenzene sulfonic acid (TNBS) method, the free amino groups produced by whey fermentation were identified. In foil-wrapped test tubes, 125 µL of material was combined with 1 mL of 0.21 M phosphate buffer, pH 8.2. The tubes were kept at 50 °C in the dark for one hour. After 60 min, the reaction was finished by adding 2 mL of 0.1 N hydrochloric acid. The samples were then compared to the control in a spectrophotometer at a wavelength of 340 nm. Deionized water generated the control, applying a concentration curve of glycine (0.05 to 0.25 mg/mL).

#### 2.3.2. Peptide Separation by Tris-Tricine-SDS-PAGE

The approach suggested by Schägger and Von Jagow [18] was applied while taking González-Olivares et al. [19]’s adjustments into consideration. Using the Bradford method, the samples’ protein concentration was normalized at 150 ppm, and 20 µL of the sample were charged. Using a gel (16.5% T) made from a 30% T solution (19:1 acrylamide:bisacrylamide ratio and 5% crosslinker, Bio-Rad, Hercules, CA, USA), electrophoresis was carried out. Gel-Doc Software (Bio-Rad, Hercules, CA, USA) was used to evaluate the gels after they had been stained using Coomassie Blue G-250 (Bio-Rad, Hercules, CA, USA).

#### 2.3.3. Separation of Peptides by SEC-HPLC

A SRT-SEC-150 SEPAX Technologies, Inc (USA) column (300 mm × 7.6 mm × 5 μm) with an exclusion range of 0. 5–150 kDa was used to separate the peptides produced during whey fermentation. A 60-min isocratic separation using a mobile phase of 0.1 M KH_2_PO_4_/K_2_HPO_4_ buffer with a pH of 6.8 was performed at 0.5 mL min^−1^ of flow rate. The target analytes were detected at a wavelength of 220 nm. The analysis was conducted in an HPLC from Perkin Elmer Series 200 connected to a manual injection system (20 µL) and a UV-vis detection system (190–380 nm).

### 2.4. ACE Inhibitory Activity

With some modifications from Cushman et al. [20], the inhibitory effect of ACE-I (EC 3.4.15.1; Sigma-Aldrich, Saint Louis, USA) was assessed spectrophotometrically. Hippuric-histidyl-leucine (HHL; Sigma-Aldrich, Saint Louis, MO, USA) was dissolved at a concentration of 5 mM in sodium borate buffer (0.1 M, pH 8.3 with 0.3 M sodium chloride). Then, 100 µL of the substrate and 40 µL of the sample (AbsM) were combined with 10 µL of ACE (EC 3.4,15.1, 5.1U/mg; Sigma-Aldrich). At 37 °C, the reaction was run for 75 min. Hippuric acid was produced and quantified at 220 nm in a Power Wave XS UV-Biotek spectrometer (KC Junior software, Kansas, MO, USA) after extraction with ethyl acetate. Then it was resuspended using deionized water and measured. The same treatment was performed for a 100% of enzymatic activity sample (AbsC) prepared with 40 µL of borate buffer instead and a 0% of enzymatic activity sample (AbsB) prepared with 50 µL of borate buffer and 100 µL of the substrate (HHL). ACE inhibitory activity was calculated using the formula based on the absorbance obtained from the measurements.

### 2.5. Statistical Analysis

ACE inhibition% = [(AbsC − AbsM)/(AbsC − AbsB)] × 100

Results were analyzed by one-way ANOVA (*p* = 0.05) and a post hoc Tukey test by using the NCSS statistical software (NCSS 2007, v.0, Kaysville, UT, USA, 2007).

## 3. Results and Discussion

### 3.1. Growth during Fermentation

**Figure 1:**
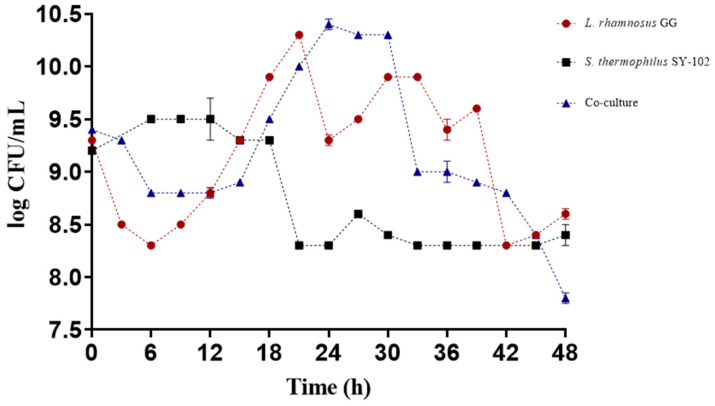
Growth during whey fermentation by *Lacticaseibacillus rhamnosus* GG ● and *Streptococcus thermophilus* SY-102 in monoculture ■ and co-culture ▲.

The fermentation results revealed their growth differed when microorganisms were grown in monoculture versus co-culture. It was observed that *L. rhamnosus* GG had better growth in monoculture. In contrast, the growth of *S. thermophilus* SY-102 did not reflect this behavior since it showed a shorter lag phase, and the logarithmic phase was reached during the first 6 h of fermentation. In contrast, *L. rhamnosus* GG showed a longer lag phase, reaching its logarithmic phase within 12 h of fermentation (Figure 1). These results are similar to those observed in other studies, where, in general, *Lactobacillus* species grow much better in monoculture compared to *S. thermophilus* SY-102 [21].

There is a synergistic effect in the co-culture, since it is known that in this type of culture the microorganisms develop a proto-cooperation relationship, releasing and exchanging metabolites necessary for optimal growth [19]. Specifically, *L. rhamnosus* GG expresses its extracellular proteinase PrtR to utilize whey proteins and provide a nitrogen source for *S. thermophilus* ssp. and itself. *S. thermophilus* ssp., on the other hand, could provide *Lactobacillus* species with formic acid, folic acid, and carbon dioxide [22,23]. In addition, it synthesizes some amino acids and expresses its cell-wall-associated proteinase (PrtS) [24].

The synergistic effect between *L. rhamnosus* GG and *S. thermophilus* ssp. impacts the growth of microorganisms and the acidification of the medium [23]. This mutualistic and proto-cooperative relationship allows for the environmental conditions necessary for the growth of both strains. However, there is competition for carbon and nitrogen sources [12].

### 3.2. pH Changes of the Medium during the Time of Fermentation

**Figure 2:**
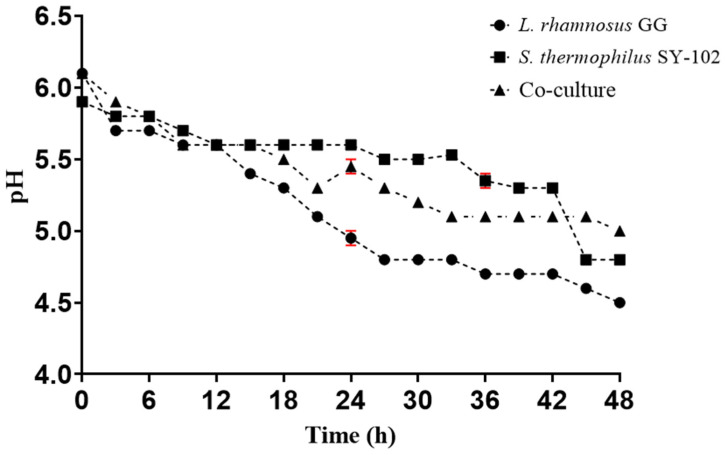
pH changes during whey fermentation by *Lacticaseibacillus rhamnosus* GG ● and *Streptococcus thermophilus* SY-102 in monoculture ■ and co-culture ▲.

The pH progressively decreased during the time of fermentation, reaching a pH of 4.5 in the case of *L. rhamnosus* GG and 4.8 for *S. thermophilus* SY-102 in monoculture. In the co-culture, the pH of the medium reached a value of 4.8 (Figure 2).

It is known that the pH decreases due to the production of lactic acid as a product of the conversion of lactose and the generation of biomass. Additionally, the differences between each system are attributed to each microorganism’s ability to use the carbon source. In some studies, it has been observed that the co-culture of these microorganisms leads to much faster medium acidification, reaching a considerably more acidic pH than a monoculture [25]. However, in this case, the pH did not decrease as drastically in co-culture. The reason for this behavior may be due to the amino groups that are released during the fermentation process, the generation of biomass itself, and the relationship of competition for the carbon source between the microorganisms.

Additionally, it is well known that the combination of *S. thermophilus* ssp. with some species of probiotics, such as *L. delbrueckii* subsp. *bulgaricus*, inhibits the rate of lactic acid production compared to monoculture [26]. Similarly, each of the microorganisms has a specific proteolytic system. This proteolytic capacity strongly influences the auxotrophies of each strain [27]. It is known that Lactobacillus species tend to adapt better to more acidic media since they modulate their intracellular pH [28]. In addition, *L. rhamnosus* GG is frequently used as a probiotic microorganism as it has a great capacity for resistance to acidic pH, colonizing, and adhering to the epithelial cells of the gastrointestinal tract [29]. On the contrary, *S. thermophilus* SY-102, despite being a lactic acid bacterium, grows much better during the first stages of fermentation, when the pH of the medium is not yet so acidic. Therefore, towards the last hours of fermentation, the culture would be dominated by *L. rhamnosus* GG.

### 3.3. Proteolysis

#### 3.3.1. Determination of Free Amino Groups by Technique of TNBS

The release of amino groups during fermentation, was different for both monoculture and co-culture systems (Table 1). The behavior observed in the monoculture of both strains was using the peptides available in the whey (time 0). Subsequently, starting from this concentration of amino groups, a decrease was observed towards 12 h of fermentation (from 367.28 ± 0.02 to 209.18 ± 0.02 µg/mL for *L. rhamnosus* GG and from 423.31 ± 0.13 to 167.52 ± 0.05 µg/mL for *S. thermophilus* SY-102). At the end of fermentation, this concentration increases again, due to the proteolytic capacity of the strains to release amino groups into the medium.

The concentration of free amino groups depends on each microorganism’s proteolytic system. It has been observed that *S. thermophilus* SY-102 in monoculture has a higher proteolytic activity in the early stages of fermentation, which decreases in the final stages [25]. However, once this microorganism satisfies its needs for amino acids such as methionine and glutamine, it releases those unnecessary amino acids and peptides into the medium [21]. In this sense, an accumulation in the medium of peptides with pyroglutamic acid and cysteine has been observed, which are not preferred by *S. thermophilus* ssp. [21]. On the contrary, *L. rhamnosus* GG requires peptides that contain cysteine, serine, arginine, proline, and glutamine [29].

In the co-culture case, a greater accumulation of amino groups could be observed towards the end of the fermentation (21 h) compared to the monocultures. The concentrations in the co-culture had a slight variation during the fermentation. However, in the last period of the logarithmic phase, they reached a maximum of 453.13 ± 0.06 µg/mL. This same behavior has been reported in other studies, where it has been observed that the concentrations of amino groups are higher in co-culture than in monocultures using the same strains [25].

Since there is a synergistic effect, the proto-cooperation interaction between the strains determines a higher concentration of amino groups [25]. Furthermore, the proteolytic systems of each strain play an important role in the initial proteolysis. In these systems, the *Lactobacillus* species have a higher initial proteolytic activity than the *Streptococcus* species [23]. This activity is determined by the protease associated with its cell wall, which initiates the hydrolysis of proteins to satisfy its auxotrophies and release peptides and free amino acids that could be used by *S. thermophilus* ssp. [30].

In contrast, the initial hydrolysis rate in *S. thermophilus* ssp. is usually very low since most hydrolysis is carried out by its system of intracellular peptidases and aminopeptidases [31]. Thus, it has been documented that the growth of *S. thermophilus* ssp. depends on the other microorganism’s metabolism when it is in co-culture. The proteolytic activity continues until the end of fermentation when the amino acids necessary for its growth have been obtained [27].

#### 3.3.2. Peptide Separation by Tris-Tricine-SDS-PAGE

**Figure 3:**
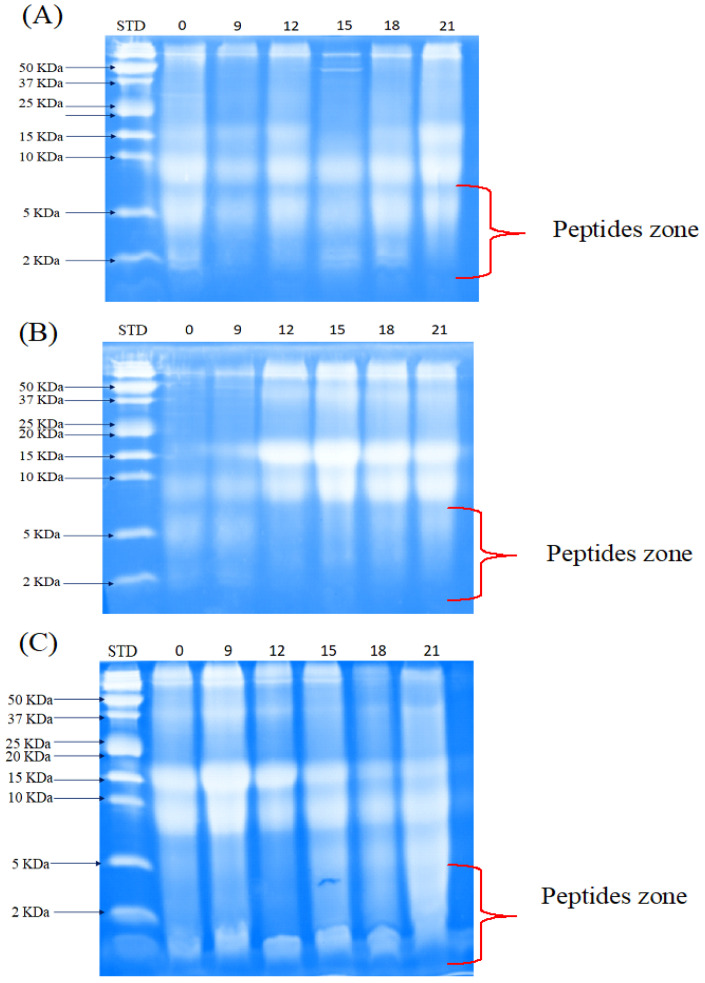
Peptide separation by SDS-PAGE in whey fermented by *L. rhamnosus* GG (**A**), *S. thermophilu*s SY-102 (**B**), and co-culture (**C**). (STD) peptides standard. Fermentation time (hours) 0–21.

Peptide separation analysis by electrophoresis showed an accumulation of peptides smaller than 10 kDa in the three systems studied (Figure 3). However, in the case of monocultures, the accumulation was higher in the culture with *L. rhamnosus* GG compared to *S. thermophilus* SY-102. This is associated with the proteolytic system of each species. In the case of *L. rhamnosus* GG, protein hydrolysis is mostly carried out through the proteinase associated with its cell wall (PrtR). Subsequently, hydrolysis continues through its system of intracellular peptidases and aminopeptidases. By contrast, at the beginning of fermentation *S. thermophilus* SY-102 shows higher activity due to its proteinase (PrtS). This activity decreases as the fermentation time increases since hydrolysis is mostly carried out by its intracellular peptidase system [25,27].

As has been pointed out, the proto-cooperation relationship between the microorganisms has a synergistic effect, which allows a greater accumulation of low molecular weight peptides in the co-culture system. In this case, the PrtR of *L. rhamnosus* GG begins protein hydrolysis, releasing amino acids necessary for *S. thermophilus* SY-102. However, the latter has specific auxotrophies, so they are satisfied through their proteolytic system. Peptides that are not needed by both species are excreted through their cell wall and released into the medium. Therefore, the peptides accumulated in the medium will be found in higher concentrations in the co-culture system.

Increasing the concentration of small peptides is interesting in this study because it has been reported that peptide size and biological activity are related. Various studies have reported peptides less than 10 kDa with antihypertensive and antidiabetic activity [7,25,32].

#### 3.3.3. Separation of Peptides by SEC-HPLC

**Figure 4:**
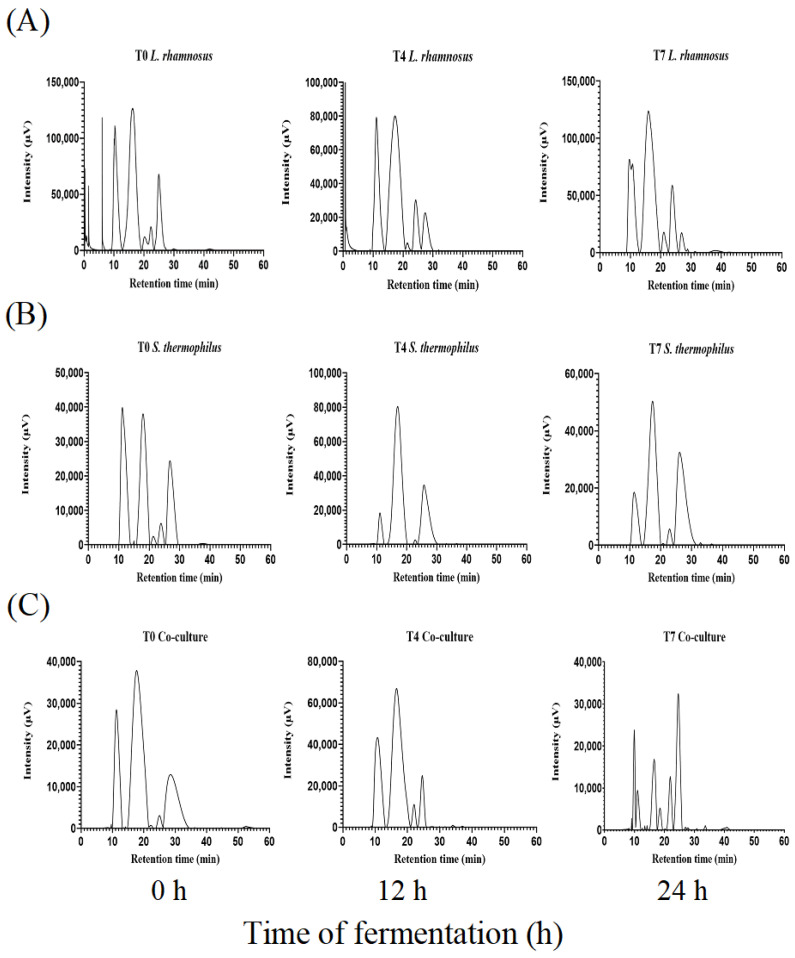
Peptide separation by SEC-HPLC of whey fermented by *L. rhamnosus* GG (**A**), *S. thermophilus* SY-102 (**B**), and co-culture (**C**). Beginning (0 h), middle (12 h), and end (21 h) of fermentation.

All fractions of peptides (beginning, middle, and end of the fermentation) were analyzed. In all three systems, a decrease in the concentration of the main globular proteins (α-lactalbumin (α-LA) and β-lactoglobulin (β-LG)) was observed. Similarly, the accumulation of small peptide fractions towards the end of fermentation (21 h) was determined. In the case of *L. rhamnosus* GG (Figure 4A), two protein fractions of 13.63 kDa and 9.43 kDa were observed at the beginning of fermentation, corresponding to whey proteins (α-LA and β-LG) with a retention time of 22 and 25 min, respectively. These fractions remained constant during the fermentation process. Towards the end of the process (21 h), the formation of a new peptide fraction of 6.9 kDa with a retention time of 27 min was detected.

In the fermentation with *S. thermophilus* SY-102 there was a decrease in the concentration of the fractions corresponding to whey proteins (Figure 4B). Likewise, two protein fractions were observed at the beginning of fermentation. The first fraction observed was 32.08 kDa, which could be the product of the association of proteins that form polymers under certain conditions of temperature and pH. The second fraction, with a molecular weight of 13.63 kDa, disappears around 21 h of fermentation. At the end of the fermentation time (21 h), two fractions of 12.15 kDa and 7.5 kDa were obtained, with a retention time of 23 and 27 min, respectively. Even though this monoculture produced low molecular weight peptide fractions, fermentation with *L. rhamnosus* GG increased their accumulation. This coincides with the results obtained in the separation by electrophoresis.

The differences in the proteolytic systems of each strain play an important role in the accumulation of peptide fractions. Intracellular peptidases reduce peptide fractions within the cell, discarding those with amino acids that are not needed [33]. However, the initial proteolysis also gives rise to free fractions in the medium. In addition, each of the strains has different auxotrophies, so the preference for these fractions will differ according to the amino acids necessary for each strain. In this sense, most of the proteolytic process of *L. rhamnosus* GG is carried out by the proteinase linked to its cell wall, PrtR. In contrast, for *S. thermophilus* ssp. this process is carried out mainly by intracellular peptidases [34].

In the case of the co-culture (Figure 4C), it was observed that the greatest accumulation of small peptide fractions appeared at the end of the fermentation process (21 h). At the beginning of the fermentation, two fractions were observed, one of nine and the other of 5.11 kDa. However, these fractions could be found naturally in the medium since the thermal process of whey treatment causes it to be partially hydrolyzed. On the other hand, a change in the concentration of the peptide fractions can be observed as the fermentation time increases. In this way, at time 7 (21 h), the accumulation of peptide fractions of 9.9 kDa was observed, with a retention time of 24.7 min.

These results coincide with evidence reported by Sebastián-Nicolás et al. [25], which they observed a greater accumulation of peptide fractions from milk fermented with *L. rhamnosus* GG and *S. thermophilus* SY-102 in co-culture. Similarly, in the co-culture fermentation, the proteolytic systems of each strain will act specifically to obtain the amino acids necessary for their metabolism. This microorganism has a series of intracellular peptidases, among which *pep*S stands out. This endopeptidase breaks the oligopeptides and releases them into the medium fractions that frequently contain hydrophobic residues and aromatic amino acids [27]. The latter has been related to an inhibition of the ACE, which leads to an antihypertensive effect [23].

### 3.4. Determination of ACE Inhibition

**Figure 5:**
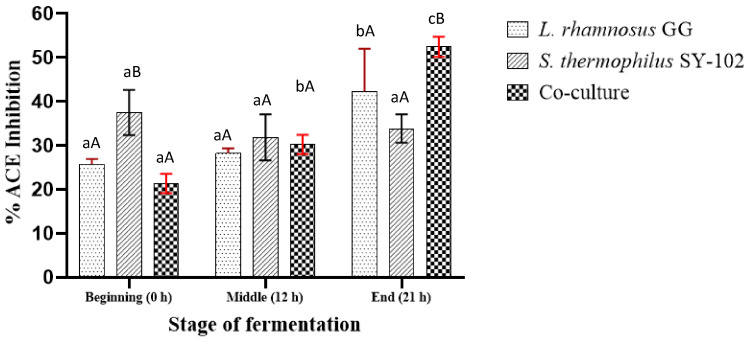
Inhibitory activity of ACE by peptidic fractions of whey fermented by *L. rhamnosus* GG, *S. thermophilus* SY-102, and co-culture. Lowercase letters compare means between times of the same fermentation system. Uppercase letters compare means between fermentation systems with the same time of fermentation.

According to the results obtained from the ACE inhibitory activity, a gradual increase in ACE inhibition was observed for the case of *L. rhamnosus* GG in monoculture. At the initial stage of fermentation (Figure 5), 25.81% of inhibition percentage was observed. Subsequently, this percentage increased to 28.23% at 12 h of fermentation, reaching an inhibition percentage of 42.34% at the end of 21 h. It is known that this microorganism needs aromatic amino acids at the beginning of fermentation to cover its metabolic needs [35]. This could explain the small increase in ACE inhibition towards the end of the first 12 h fermentation. However, once it has enough aromatic amino acids, it releases unnecessary ones into the medium, increasing the percentage of inhibition towards 21 h of fermentation.

In the case of *S. thermophilus* SY-102 in monoculture, a decrease in the percentage of ACE inhibition from 37.5% to 31.85% was observed. Subsequently, this percentage increases to 33.87% after 21 h of fermentation, reflecting a slight increase in inhibitory activity. It is known that *S. thermophilus* ssp. does not prefer aromatic amino acids, and peptide accumulation is imminent. However, the fact that it did not reach a higher inhibition percentage could be related to the results observed in its growth, which was not superior to *L. rhamnosus* GG. According to the electrophoresis results, its proteolytic capacity to produce low molecular weight peptides was lower than that of *L. rhamnosus* GG monoculture and co-culture. This could be related to a lower ACE inhibitory activity, as the accumulation of peptides containing aromatic amino acids was not encouraged during fermentation.

An opposite effect could be observed from the co-culture. The percentage of ACE inhibition gradually increased from 21.37% to 52.42% by 21 h of fermentation. This result is consistent with those observed in the proteolytic profile of the co-culture, in which there was a higher concentration of free amino groups and peptide fractions of low molecular weight. It has been reported that symbiotic growth in co-culture systems leads to a wide variety of bioactive peptides and a higher concentration of them. The differences in the proteolytic systems of the strains favor the accumulation of peptides in the medium and increase the antihypertensive activity [25,36].

Various studies have pointed out the close relationship between the structure and bioactivity of peptides [37,38]. In the case of ACE inhibition, it has been pointed out that inhibitors contain hydrophobic amino acids in their structure [23]. Specifically, antihypertensive activity is strongly associated with the sequence present at the C-terminal site [5]. Thus, peptides with amino acid residues such as tryptophan, proline, phenylalanine, and isoleucine tend to be more potent in ACE inhibition by interacting with its active site [39].

Using microorganisms in co-culture to ferment certain protein matrices, such as whey, is an opportunity to produce antihypertensive peptides. This increases the possibility of generating fermented dairy products with functional potential, especially due to the advantages of using microorganisms.

## 4. Conclusions

The combination of both lactic acid bacteria studied in whey fermentation increases the growth of both microorganisms through a proto-cooperative relationship. Similarly, a co-culture system favors the strains’ proteolytic activity, increasing the concentration of low molecular weight peptide fractions. Small peptides and, most likely, aromatic amino acids in the structure could explain the antihypertensive activity observed in this study. Likewise, this activity increases when using a co-culture system compared to a monoculture.

Microbial fermentations involving the proto-cooperation of lactic acid bacteria represent a promising source of peptides with health-promoting properties. The sequences derived from the fractionation of whey proteins represent an important field of study to evaluate their role in various pathologies and diseases.

## Figures and Tables

**Table 1:** Concentration of free amino groups from the fermentation of whey with *L. rhamnosus* GG, *S. thermophilus* SY-102, and both microorganism in co-culture. The mean ± standard deviation (SD) expressed the results of three replicates.

	Free Amino Groups μg/mL		
Time of Fermentation (h)	*L. rhamnosus* GG	*S. thermophilus* SY-102	*L. rhamnosus* GG + *S. thermophilus* SY-102
0	366.91 ± 0.02 ^aAB^	463.43 ± 0.13 ^aA^	226.59 ± 0.15 ^aAB^
9	277.67 ± 0.01 ^abA^	264.63 ± 0.02 ^bA^	285.28 ± 0.13 ^aA^
12	208.97 ± 0.02 ^abcA^	167.34 ± 0.05 ^bcA^	363.32 ± 0.02 ^aB^
15	239.85 ± 0.02 ^abcA^	255.06 ± 0.00 ^bA^	253.76 ± 0.20 ^aA^
18	277.23 ± 0.07 ^abA^	350.60 ± 0.03 ^abdA^	268.97 ± 0.17 ^aA^
21	293.54 ± 0.05 ^abA^	323.54 ± 0.04 ^abAC^	452.67 ± 0.06 ^aB^

Lowercase letters compare means between times of the same fermentation system. Uppercase letters compare means between fermentation systems with the same time of fermentation. According to Tukey’s test, the same letter did not present a significant difference (*p* < 0.05).

## Data Availability

All research data is available and can be requested directly from the corresponding author at lgonzales@uaeh.edu.mx.
